# Enabling Droplet Functionality on Anisotropic Ratchet Conveyors

**DOI:** 10.3390/mi8120363

**Published:** 2017-12-16

**Authors:** Hal R. Holmes, Ana E. Gomez, Karl F. Böhringer

**Affiliations:** 1 Department of Bioengineering, University of Washington, Seattle, WA 98105, USA; hrholmes@uw.edu (H.R.H.); anag3@uw.edu (A.E.G.); 2 Department of Electrical Engineering, University of Washington, Seattle, WA 98195, USA

**Keywords:** droplet transport, microfluidics, vibrations, contact line oscillation, asymmetric surfaces, anisotropic ratchet conveyor

## Abstract

Anisotropic ratchet conveyors (ARCs) are a recently developed microfluidic platform that transports liquid droplets through a passive, microfabricated surface pattern and applied orthogonal vibrations. In this work, three new functionalities are presented for controlling droplet transport on the ARC system. These devices can pause droplet transport (ARC gate), decide between two pathways of droplet transport (ARC switch), and pass droplets between transport tracks (ARC delivery junction). All devices function solely through the modification of pinning forces acting on the transported droplet and are the first reported devices that can selectively control droplet timing and directionality without active (e.g., thermal, electrical, or magnetic) surface components.

## 1. Introduction

**Figure 1:**
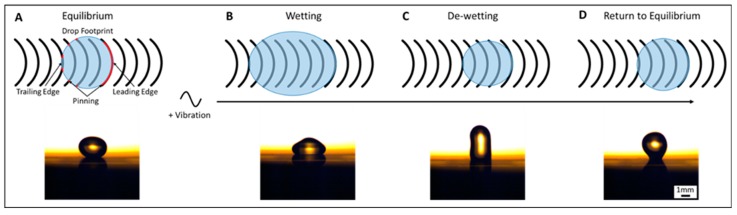
Principles of anisotropic ratchet conveyor (ARC) functionality. ARC systems transport droplets through an anisotropic surface pattern composed of periodically occurring curved rungs (black) defined by a hydrophobic background (white). This asymmetric geometry creates a difference in pinning between leading and trailing edges of the contact line or “footprint” of the droplet (**A**). Applied orthogonal vibrations induce the contact line to oscillate between wetting, de-wetting and equilibrium states (**B**–**D**). This combination results in a net force through each vibration cycle that transports droplets.

Anisotropic ratchet conveyors (ARCs) are a type of digital microfluidic (DMF) system that can transport an individual liquid droplet or many droplets in parallel through a passive micropatterned surface and applied orthogonal vibrations. The functionality of ARC devices comes from two primary features: (1) an anisotropic surface pattern of periodically occurring curved structures or “rungs,” and (2) oscillation of the contact line or “footprint” of the droplet on the substrate, induced by the applied orthogonal vibrations [1]. The asymmetry of the surface pattern creates a difference in pinning forces between leading and trailing edges of the droplet. The applied vibrations cycle the contact line between wetting, de-wetting, and equilibrium phases. This combination produces a net force in the direction of the leading edge, which essentially causes the droplet to take a step through each vibration cycle (Figure 1) [1,2,3]. 

While ARCs do not offer the robust programmability available to electrowetting-on-dielectric (EWOD) or dielectrophoresis (DEP)-based DMF systems [4,5,6], this platform provides the ability to handle liquid droplets with a passive surface pattern and a globally applied sinusoidal vibration (e.g., a speaker). This configuration allows for droplets to be driven with a single signal source, which substantially simplifies the circuitry and programming required to operate an ARC system. Like EWOD and DEP systems, the ability of ARCs to handle liquid in the form of discrete droplets can reduce required sample volumes and reagent quantities compared to continuous flow devices. Droplets also provide a form of ‘compartmentalization’, wherein the contents of each droplet are individually isolated, preventing undesirable interactions between samples or reagents [4].

The simple microelectromechanical systems (MEMS)-based fabrication process allows for high-throughput manufacturing of ARC devices, which could provide for inexpensive ARC chips with integrated MEMS components or electronic sensors. Such a system could fill the niche for diagnostic or analytic applications that require more process control or measurement accuracy than paper-based or passive microfluidic systems [7,8]. The simplicity of this system makes it a good candidate for field-ready or point-of-contact (where the sample is first encountered in the field) tests, potentially enabling a point-of-contact platform with improved clinical utility [9,10,11], or for molecular (nucleic acid) assays that are less expensive and more easily deployable [12,13,14]. Furthermore, ARCs could also provide a useful research tool, such as in applications for automating protein [15] or nucleic acid [16] purification. 

However, before any applications for an automated ARC platform can be realized, the functional toolbox available to ARC systems must be expanded. Thus, we have developed three new modules for the ARC system: (1) ARC gates that can selectively pause droplet transport, (2) ARC switches that can select the direction of droplet transport between two paths, and (3) ARC delivery junctions that can controllably deliver droplets on a convergent path. In electrowetting systems, these functions are innately enabled by the position of electrodes, with respect to the droplets, being activated [4,17,18]. On ARC systems, functionality is dictated by the design of the passive surface pattern. Therefore, each droplet function on ARC systems must be enabled with a specific design strategically placed on chip. The following sections will demonstrate how the design of the surface pattern on these ARC modules pairs with the applied vibrations to enable essential functions for automated liquid handling processes on ARC systems.

## 2. Materials and Methods 

**Figure A1:**
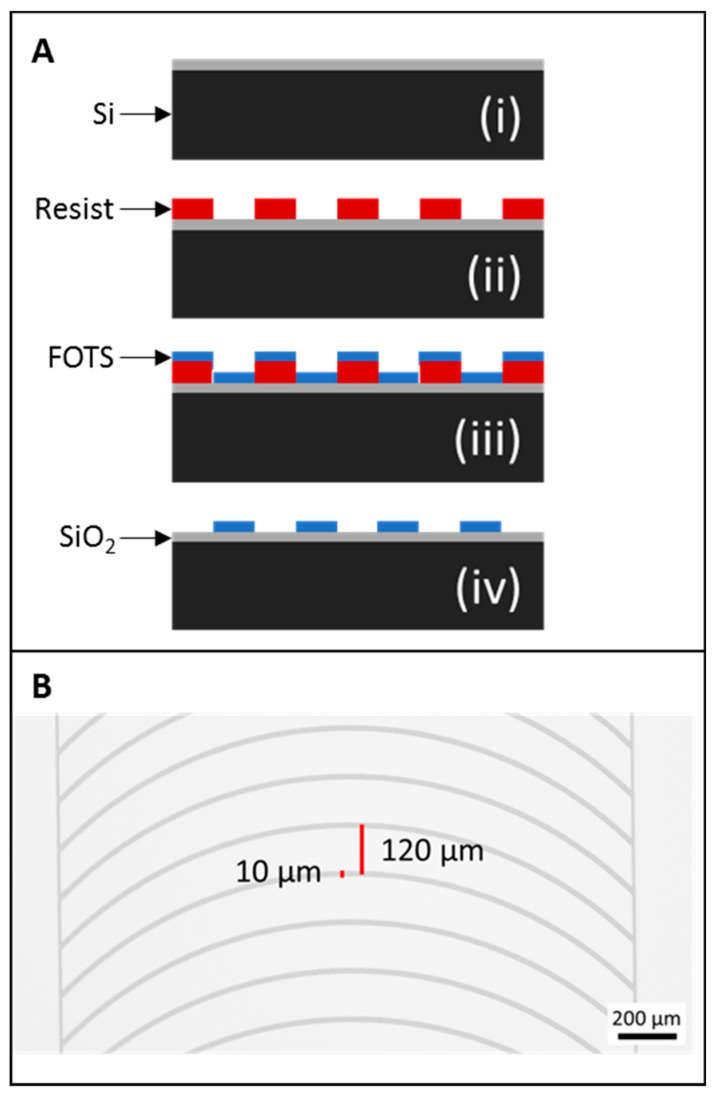
ARC fabrication and duty cycle. SiO_2_-FOTS ARCs are fabricated on a silicon wafer with an SiO_2_ surface layer (**A**-**i**). The ARC design is patterned with photoresist (**A**-**ii**) and the wafer is coated with FOTS (**A**-**iii**). Stripping the resist reveals the hydrophilic SiO_2_ pattern (**A**-**iv**). Rung duty cycle is defined as the width of the rungs (10 µm) divided by the period between rungs. For example, a 120 µm period (**B**) provides a duty cycle of 8.3%.

All ARC devices used in this work are fabricated by patterning an oxidized silicon wafer with photoresist. Exposed regions of the wafer are coated with fluorooctyltrichlorosilane (FOTS). The resist is then stripped with acetone, revealing hydrophilic (contact angle < 5° [19]) silicon dioxide (SiO_2_) rungs defined by the hydrophobic (contact angle of approximately 105° [19]) FOTS background (Figure A1). At rest, both leading and trailing edges exhibit a contact angle near a native FOTS surface, but the contact angles at the two edges differ during forced vibration as a result of the asymmetric ARC pattern [20]. Images of ARC designs are captured prior to resist stripping and superimposed for clarity, as the final ARC devices in this work are invisible to the naked eye. 

For all experiments in this work, 10 µL droplets of deionized water (diH_2_O) were driven on ARC substrates with sinusoidal vibrations produced by a function generator and an electromagnetic motor. Target frequencies were applied through the programming of the function generator. The acceleration amplitude of applied vibrations was measured with a laser-Doppler vibrometer, and images of moving droplets were captured with a high-speed camera. Measurements of droplet edge displacement were performed in MATLAB (The MathWorks, Natick, MA, USA) using custom scripts. All numeric data is presented as mean ± standard deviation.

## 3. Results and Discussion

ARC tracks are characterized by the minimum acceleration amplitude at which the substrate must be vibrated to initiate droplet transport (ARC threshold). This threshold is known to be dependent on volume and material properties of the droplet (e.g., surface tension) and the interaction of the droplet footprint with the ARC surface pattern [19,20]. Similarly, devices presented in this work are characterized for droplets 10 µL in volume and different behaviors are to be expected if the size or liquid of the droplet are sufficiently altered. However, the scaling of droplet behavior on ARC devices is predictable [20], and it is expected that scaling of the feature size for these devices could in turn accommodate a wider range of sizes and liquid properties. 

### 3.1. Effects of Duty Cycle

**Figure 2:**
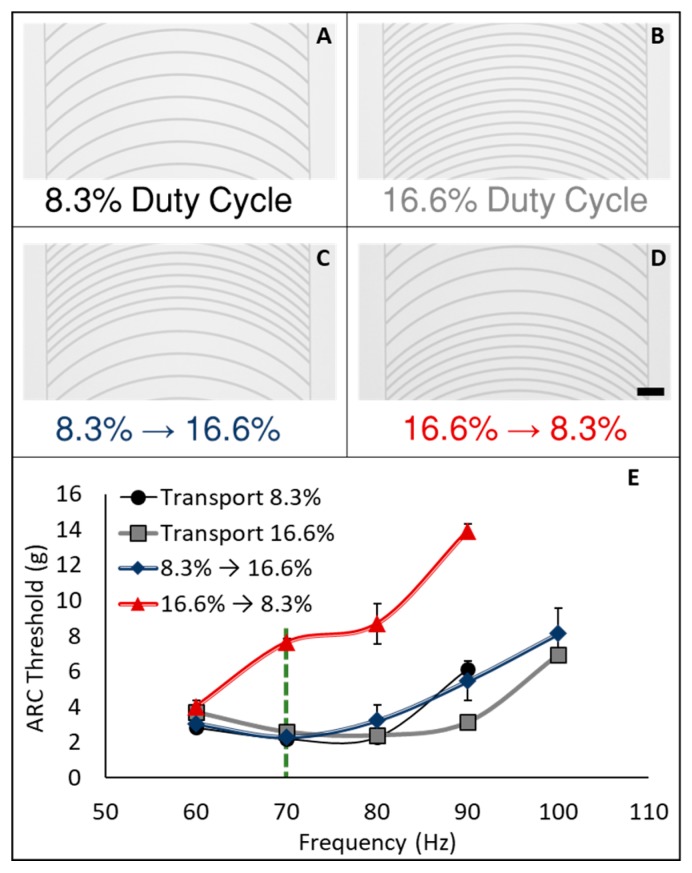
Rung duty cycle modulates ARC threshold. The ARC threshold for droplet transport (10 µL diH_2_O) was measured on ARC tracks with 8.3% (**A**) and 16.6% (**B**) duty cycles, and transitions from 8.3% to 16.6% (**C**) and 16.6% to 8.3% (**D**). Only the transition from 16.6% to 8.3% required a significantly higher ARC threshold at frequencies above 60 Hz (**E**). The dotted green line indicates 70 Hz response used in subsequent experiments with ARC gates. Scale bar = 200 µm.

**Figure A2:**
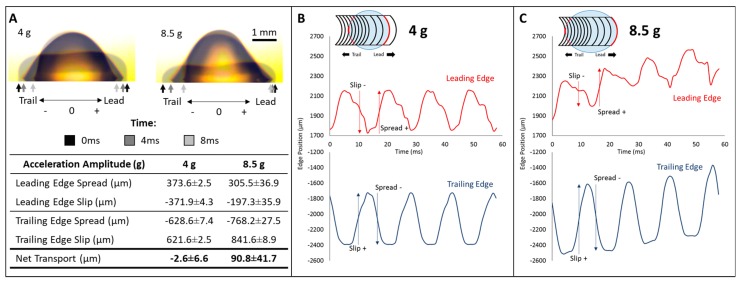
Increased trailing edge mobility reduces slip at leading edge. De-wetting sequence (**A**—figure overlay) demonstrates the difference in droplet response when vibrated on a 16.6% to 8.3% duty cycle transition at 4 g and 8.5 g. Measurements of droplet edges (**A**—table) indicate slip (de-wetting) and spread (wetting) is the same for both edges at 4 g. Raising the vibration amplitude to 8.5 g increased the spread of the trailing edge, but actually reduced the spread of the leading edge. However, this resulted in a lower slip at the leading edge and higher slip at the trailing edge (compared to spread), which provided for droplet transport. Real-time positions of droplet edges (with respect to the droplet center at 0) at 4 g (**B**) and 8.5 g (**C**) demonstrate how these observed differences in slip and spread translate to net transport at 8.5 g. Note that at the leading edge, spread is in the positive direction (direction of net transport) and slip is in the negative direction, conversely, at the trailing edge, spread is in the negative direction while slip is in the positive direction. Additionally, slip is defined as the distance from maximum to minimum wetting, while spread is the distance from minimum wetting to maximum wetting in the next half of the cycle.

On ARC patterns used in this work, we define rung duty cycle as the width of the hydrophilic rung divided by the hydrophobic period (center to center distance) between rungs (Figure A1). ARC designs used here consisted of 10 µm wide rungs with a radius of 1000 µm and a period of 60 µm or 120 µm, providing for a duty cycle of 16.6% or 8.3%, respectively (Figure 2). The ARC threshold of the SiO_2_-FOTS tracks was first determined over a range from 60 to 100 Hz (Figure 2E). We observed that the ARC threshold profiles, although not identical, were relatively similar on tracks with both 8.3% and 16.6% duty cycles. Additionally, the transition from 8.3% to 16.6% duty cycle also demonstrated an overlapping ARC threshold profile. However, the ARC threshold for the transition from 16.6% to 8.3% duty cycle exhibited a unique profile with significantly higher vibration thresholds above 60 Hz. The observed increase in ARC threshold is due to the combination of increased pinning on the higher duty cycle region (trailing edge—facing the direction opposite of transport) and increased slip (de-wetting) on the lower duty cycle region (leading edge—facing the direction of transport—Figure A2). Essentially, the high duty cycle region creates more drag on the trailing edge, requiring a larger vibration amplitude to initiate transport.

### 3.2. Anisotropic Ratchet Conveyor (ARC) Gates

**Figure A3:**
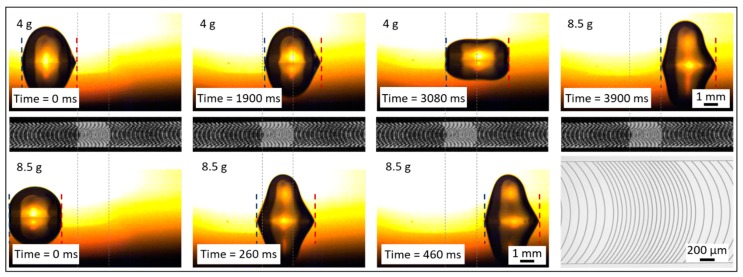
Droplet gate selectively passes droplets. Droplets (10 µL) driven with a 70 Hz vibration of 4 g stopped at the gate (design—bottom right) until a vibration of 8.5 g was applied (**top sequence**). Droplets driven with a vibration of 8.5 g moved through the gate without stopping (**bottom sequence**). Center panels and gray dotted lines depict the track and gate with respect to the droplets. Red and blue dotted lines depict the leading and trailing edges of the droplets, respectively.

The effects of duty cycle transitions were then employed to enable “ARC gates”, which can selectively pause droplet transport based on the signal of the applied vibrations. Droplet gates were developed by nesting a region with a higher (16.6%) duty cycle within a track composed of a lower (8.3%) duty cycle. Droplets driven by vibrations below the ARC threshold for the gate will pass through the transition from low to high duty cycle, but will pause on the transition from 16.6% to 8.3% duty cycle. When the vibration signal is increased above the ARC threshold for the gate, droplet transport will resume. Additionally, if a droplet is driven with a vibration above the ARC threshold for the gate before entering the gate, then it will pass through without stopping (Figure A3).

**Figure 3:**
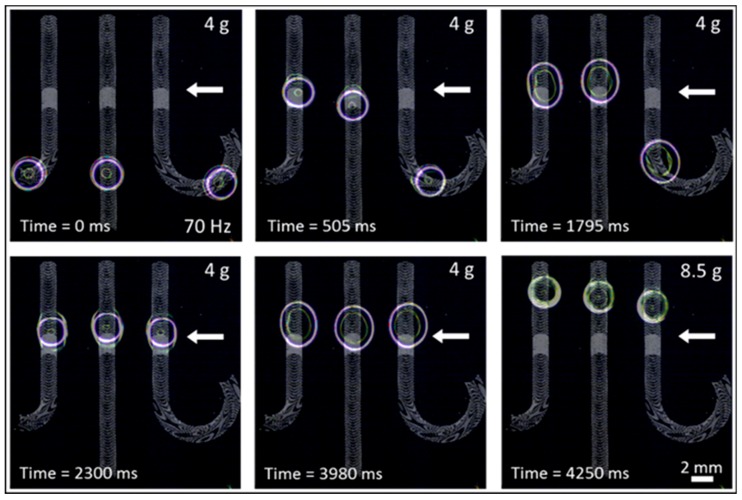
Droplet synchronization with ARC gates. Droplets transported on unique ARC paths with vibrations below the threshold of the ARC gate will pause at the transition from 16.6% to 8.3% duty cycle (indicated by the white arrow). Droplets will remain indefinitely at this position in the ARC gate, which allows droplets on all transport paths to line up (ARC patterns are superimposed in grey). Increasing the vibration signal above the gate threshold continues droplet transport in a tight distribution.

**Figure A4:**
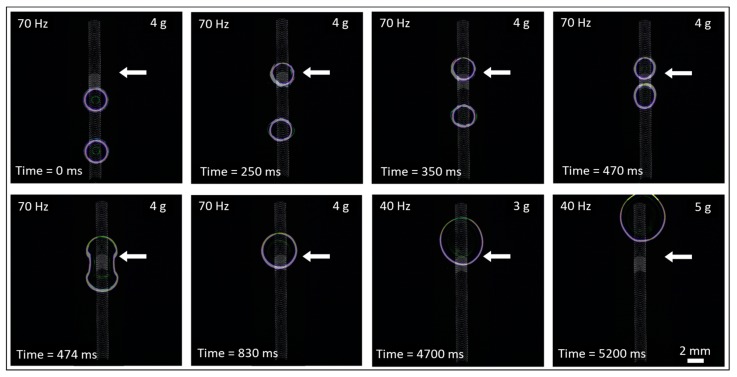
Droplet gates can mix droplets of the same volume. Two droplets (10 µL) are transported on an ARC track with a droplet gate. Both droplets are transported, maintaining their initial separation, until the first droplet comes to the droplet gate. The gate holds this droplet in place until the second droplet comes in contact. The droplets spontaneously merge, forming a larger droplet. The larger droplet is then held in place by the droplet gate until sufficient vibration amplitude is applied (5 g). Note that the larger droplet has a lower natural frequency, which requires a lower vibration frequency for transport (40 Hz). The track design is depicted by the overlay (gray) and the white arrows indicate where the transition from the 16.6% to 8.3% duty cycle occurs.

Stopping droplets on an ARC chip was previously achievable by turning off the vibration signal. However, this would stop all droplets being transported on a chip. ARC gates provide the ability to pause a single droplet without affecting the transport of other droplets on chip. For example, Figure 3 (also shown in Video S1) demonstrates how droplets with unique transport paths can be synchronized with ARC gates. On this chip, three droplets, each on a distinct ARC path, are transported by vibrations below the ARC threshold for the gate. The transport of each droplet will be paused once it reaches the gate. This allows for droplets on longer paths, such as the droplet on the left, or droplets that are performing processes elsewhere on chip to continue their transport. Once all three droplets have lined up on the gates, the vibration amplitude is increased, resuming the transport of all droplets in a tight distribution. In addition to synchronization, these gates can also be applied on an ARC system to hold droplets over a detection region or sensor, controllably mix droplets in the same transport path (Figure A4 and Video S2), and control the timing or sequencing of a droplet on chip.

### 3.3. Anisotropic Ratchet Conveyor (ARC) Switches

**Figure 4:**
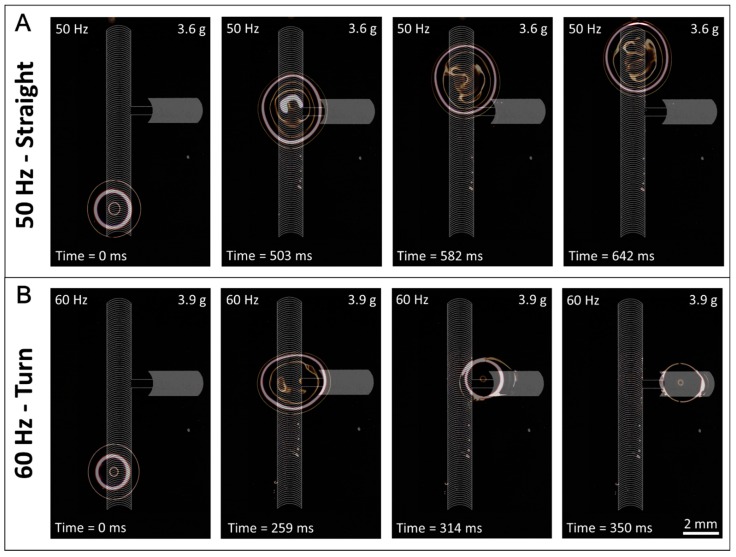
ARC switches can select direction of droplet transport. Image sequence shows droplets transported on an ARC switch having a main track of an 8.3% duty cycle with a perpendicular track of a 16.6% duty cycle. Droplets transported at 50 Hz and 3.6 g (**A**) contact the perpendicular track but move straight through the intersection. Vibrations of 60 Hz and 3.9 g (**B**) provide sufficient wetting and aspect ratio to turn the droplets at the intersection.

**Figure A5:**
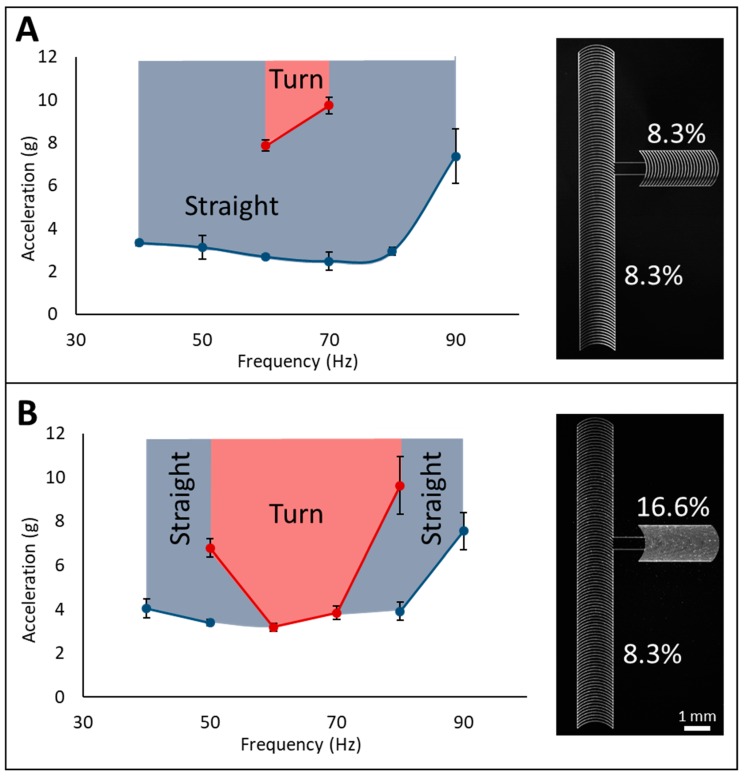
Perpendicular intersection enables ARC switch. The ARC thresholds for transporting droplets straight through or turning at the intersection were measured for switches having main and perpendicular tracks with 8.3% duty cycle (**A**) and a main track of 8.3% with a perpendicular track of 16.6% duty cycle (**B**). The increased pinning of the higher duty cycle perpendicular track enabled droplets to turn at much lower vibration amplitudes. Blue regions correspond to vibration parameters that drive the droplet straight through the intersection, while red regions correspond to parameters that turn the droplet at the intersection.

A transition in duty cycle changes the balance of pinning forces along one dimension of the droplet (between the leading and trailing edges). To understand how this balance of forces responds to changes in two dimensions, we added a second track perpendicular to the main track. In this case, pinning forces act on the leading and trailing edges of the droplet like a normal ARC track, but when the droplet reaches the perpendicular track, pinning forces also act on one side of the droplet. We found this simple combination provides an intersection, or switch, that can dictate the direction of droplet transport based on the applied vibration signal (Figure 4). Previously, switches on ARC systems had been realized through pairing with electrowetting [21], but the switches presented here are the first to provide the capability of controlling droplet directionality with no active surface components. The threshold profile for ARC switches was determined as previously discussed. However, switches have two thresholds—(1) the vibration required for a droplet to be transported through the intersection on the main track (straight—Video S3) and (2) the vibration required for the droplet to turn onto the perpendicular track (turn—Video S4). Expectedly, turning is more efficient when the duty cycle of the perpendicular track is higher than that of the main track (Figure A5).

**Figure A6:**
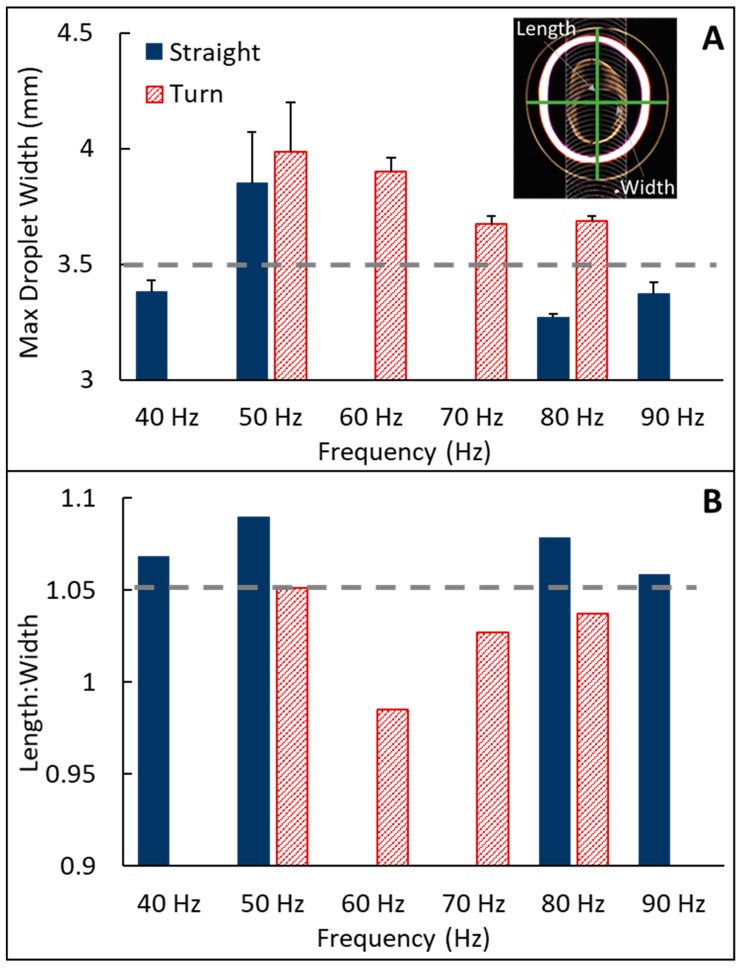
Turning droplets depends on droplet width and aspect ratio. The length and width (insert) of droplets during maximum wetting were measured for switches with a 16.6% duty cycle perpendicular track (8.3% main track). These data indicate that two conditions must be met for a droplet to turn: the width of the droplet during wetting must be large enough to contact the perpendicular track (this distance is indicated by the dotted grey line—(**A**), and the aspect ratio (**B**) must be sufficient for pinning forces on the right edge of the droplet to dominate.

Data gathered from videos of droplets on the switches (shown in supplementary information) also indicate that two conditions must be met for turning to occur. The first condition is that the droplet edges must expand enough to contact the perpendicular track (Figure A6A). Although this condition is somewhat trivial, it suggests that these switches could innately sort droplets based on volume, as small droplets would be unable to sufficiently expand to contact the perpendicular track. The second condition is that the aspect ratio (length of the droplet in the direction of the main track divided by the width of the droplet perpendicular to the track) during wetting must be low enough (i.e., the droplet must be wide enough) for the pinning forces of the perpendicular track to dominate and change the direction of the droplet (Figure A6B). Furthermore, the shape of the droplets was always circular or elliptical and the formation of additional nodes was not observed. This indicates that a change in vibration mode [22,23] is not responsible for these observations. 

### 3.4. Anisotropic Ratchet Conveyor (ARC) Delivery Junctions

**Figure A7:**
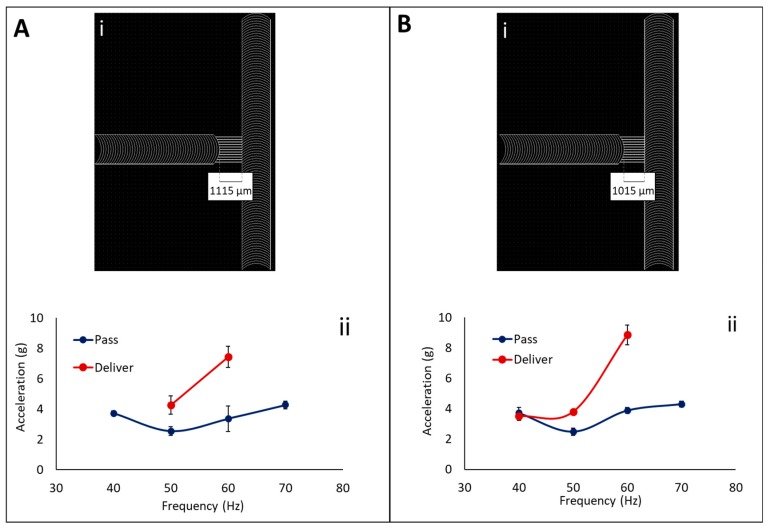
Delivery junctions can transfer droplets between tracks without impeding transport. Pass and delivery thresholds for delivery junctions with (**A**) 1115 µm and (**B**) 1015 µm separation between the terminating track and the main track were investigated. Thin hydrophilic guides (**Ai**,**Bi**) provide connectivity between the terminating track and main track without impeding transport of droplets on the main track. ARC thresholds (**Aii**,**Bii**) to pass a droplet on the main track by this junction and deliver a droplet across this junction were characterized for 10 µL droplets.

Passing droplets from one track to another is an important capability that is necessary for mixing droplets processed on alternate paths or derived from different sources. However, this function is non-trivial with ARC systems because simply merging two paths can create a local concentration of pinning forces (hydrophilic regions) that can impede transport through these regions (i.e., droplets get stuck). In order to provide this functionality to our ARC system, droplet junctions were developed that can deliver a droplet to an adjacent track without compromising transport of droplets on either track. This was accomplished by connecting the two tracks with hydrophilic guides. The spacing of these guides was adjusted to provide for delivery of a droplet from the terminating track without compromising the transport of droplets along the main track (i.e., if the guides are too close together droplets will get stuck at the junction, but if they are too far apart the droplet on the terminating track cannot be delivered to the main track). We hypothesize that the hydrophilic guides promote delivery from the terminated track by wicking or pulling the droplet edge towards the main track. When paired with the correct vibration signal, pinning forces on the main trail will overtake the droplet from the terminating track. Due to these interactions of droplets from different directions, two thresholds also exist for the delivery junctions: (1) Pass—the vibration amplitude required for the droplet to travel on the main track and pass the hydrophilic guides without getting stuck, and (2) Deliver—the vibration amplitude required to transfer a droplet from the terminating track onto the main track (Figure A7). Interestingly, the relationship of the pass and deliver thresholds appears quite similar to that for ARC gates. 

**Figure 5:**
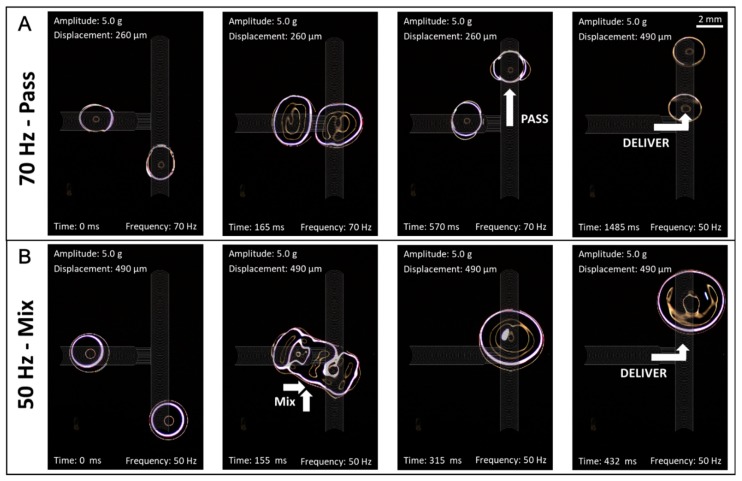
Delivery junctions can transfer and mix droplets. Droplets on a terminated track can be delivered to a main track with a delivery junction. At this intersection droplets can (**A**) pass by each other or (**B**) merge depending on the applied vibration signal. Most importantly, delivery junctions accomplish this functionality without impeding transport of droplets on the main track.

Additionally, we discovered that these thresholds also provide distinct droplet behaviors when two droplets meet at this junction. Under vibration parameters that meet the conditions for passing the junction but not delivery, a droplet will be held at the junction while the droplet on the main track passes by. The droplets will remain separate, even though they appear to nearly touch (supplementary videos), and the droplet at the junction can be later delivered with appropriate vibration signal (Figure 5A and Video S5). However, if vibration parameters for delivery are applied, the droplets will merge at the junction, and the merged droplet will be subsequently delivered to the main track (Figure 5B and Video S6). Thus, this seemingly simple configuration presents a large variety of possible functions that can be performed through strategic selection of vibration parameters.

## 4. Conclusions

ARCs are a recently developed microfluidic platform that transports liquid droplets through a passive surface pattern and orthogonal vibrations. The facile fabrication and operation of ARC devices shows much potential to meet applications in low-cost diagnostic and analytic applications. In this work, we demonstrate new expansions to the ARC functional toolbox with the development of ARC gates, switches, and delivery junctions. All three modules derive their utility by changing the balance of pinning forces between edges of a transported droplet, either in one or two dimensions. ARC gates can controllably pause droplet transport through an increase in pinning forces at the trailing edge of a droplet, ARC switches provide control over droplet direction at an intersection by applying pinning forces at a side edge of the droplet, and ARC delivery junctions use hydrophilic guides to transfer droplets between tracks without impeding transport. In short, this system combines a simple hardware platform with a sequence of sine wave signals. Expanding on this work, future ARC devices could perform complex protocols that require conditional processing steps, or sequential regimens. 

## 5. Patents

This work resulted in US Provisional Patent Applications No. 62/281,879 and No. 62/302,948 that have been converted into an International Patent Cooperation Treaty (PCT) Patent (PCT/US2017/014529) entitled “Contact-Line-Driven Microfluidic Devices and Methods”. 

## Supplementary Materials

The following are available online at www.mdpi.com/2072-666X/8/12/363/s1, Video S1. Droplet synchronization on ARC gates; Video S2. Merging droplets on ARC gates; Video S3. ARC switch straight direction; Video S4. ARC switch turn direction; Video S5. Passing droplets on ARC delivery junctions; Video S6. Merging droplets on ARC delivery junctions.
